# NAD-linked mechanisms of gene de-repression and a novel role for CtBP in persistent adenovirus infection of lymphocytes

**DOI:** 10.1186/s12985-019-1265-y

**Published:** 2019-12-21

**Authors:** Megan L. Dickherber, Charlie Garnett-Benson

**Affiliations:** Charlie Garnett-Benson, Department of Biology, Georgia State University, 161 Jesse Hill Jr. Dr, Atlanta, GA 30303 USA

**Keywords:** Adenovirus, Species C, Viral persistence, Human lymphocytes, Reactivation, CtBP, Metabolism

## Abstract

**Background:**

Adenovirus (AdV) infection is ubiquitous in the human population and causes acute infection in the respiratory and gastrointestinal tracts. In addition to lytic infections in epithelial cells, AdV can persist in a latent form in mucosal lymphocytes, and nearly 80% of children contain viral DNA in the lymphocytes of their tonsils and adenoids. Reactivation of latent AdV is thought to be the source of deadly viremia in pediatric transplant patients. Adenovirus latency and reactivation in lymphocytes is not well studied, though immune cell activation has been reported to promote productive infection from latency. Lymphocyte activation induces global changes in cellular gene expression along with robust changes in metabolic state. The ratio of free cytosolic NAD^+^/NADH can impact gene expression via modulation of transcriptional repressor complexes. The NAD-dependent transcriptional co-repressor C-terminal Binding Protein (CtBP) was discovered 25 years ago due to its high affinity binding to AdV E1A proteins, however, the role of this interaction in the viral life cycle remains unclear.

**Methods:**

The dynamics of persistently- and lytically-infected cells are evaluated. RT-qPCR is used to evaluate AdV gene expression following lymphocyte activation, treatment with nicotinamide, or disruption of CtBP-E1A binding.

**Results:**

PMA and ionomycin stimulation shifts the NAD^+^/NADH ratio in lymphocytic cell lines and upregulates viral gene expression. Direct modulation of NAD^+^/NADH by nicotinamide treatment also upregulates early and late viral transcripts in persistently-infected cells. We found differential expression of the NAD-dependent CtBP protein homologs between lymphocytes and epithelial cells, and inhibition of CtBP complexes upregulates AdV E1A expression in T lymphocyte cell lines but not in lytically-infected epithelial cells.

**Conclusions:**

Our data provide novel insight into factors that can regulate AdV infections in activated human lymphocytes and reveal that modulation of cellular NAD^+^/NADH can de-repress adenovirus gene expression in persistently-infected lymphocytes. In contrast, disrupting the NAD-dependent CtBP repressor complex interaction with PxDLS-containing binding partners paradoxically alters AdV gene expression. Our findings also indicate that CtBP activities on viral gene expression may be distinct from those occurring upon metabolic alterations in cellular NAD^+^/NADH ratios or those occurring after lymphocyte activation.

## Background

Adenovirus infection is ubiquitous in the human population, and the species C subgroup (AdV-C1, 2, 5, and 6) is the most widespread of the viruses. Species C AdVs cause acute infection in the respiratory and gastrointestinal tracts [1–4]. In addition to causing lytic infections in epithelial cells, adenoviruses have the ability to persist in a non-lytic state in mucosal lymphocytes [2, 5–11]. AdV-C infections occur predominantly in the very young, and consequently nearly 80% of children contain viral DNA in the lymphocytes of their tonsils and adenoids [1–4]. These infections can be life-threatening for immunocompromised pediatric transplant patients, and those receiving allogeneic hematopoietic stem cell transplants (allo-HSCT) are at significant risk for developing disseminated adenovirus disease. Although these infections and resulting disease can be initiated through de novo exposure to the virus, the predominant cause in severely immunocompromised patients is endogenous reactivation of AdV-C, types 1, 2, and 5 [3]. The AdV-related post-transplantation mortality for these patients is estimated to be between 3.2 and 6.0%, potentially affecting more than 100 children per year in the U.S. [3, 12, 13]. There is currently no medical intervention to protect against AdV reactivation, or FDA-approved treatment for AdV disease, and the mechanisms that allow the virus to persist and induce reactivation are almost entirely unknown [14, 15].

Persistent AdV infections last for long periods of time following resolution of the initial lytic infection, and the virus can be intermittently detected in fecal samples for months to years after symptoms have abated [16]. Persistent infections in lymphocytes have been reported to exhibit a range of repressed states, from truly latent (with no production of infectious particles) to a “smoldering” infection with low viral yield [2, 8]. Immunoactivation of tonsillar lymphocytes has been shown to reactivate latent AdV, but the cell-type specific mechanisms behind this de-repression have not been studied [2]. B and T lymphocytic cell line models of persistent infection have been established that exhibit long-term persistent AdV infections marked by retention of high levels of viral genomes and very low viral protein expression [17, 18]. Interestingly, the persistent phase in these models has been shown to be regulated, in part, by transcriptional controls not seen in lytic infections. Several viral genes have been reported to display alternative patterns of expression when compared to lytic infections, suggesting specific programs of repression are present in persistent infections of lymphocytes [19–21].

As B and T lymphocytes transition from a resting to an activated state, they undergo dramatic shifts in gene expression and metabolism to accommodate robust proliferation and differentiation into effector cells. Programs of gene expression during both resting and activated states have been shown to be regulated in part by chromatin remodelers and co-repressors, including DNA methyltransferases (DNMTs), Class I and II histone deacetylases (HDACs), Class III HDACs (sirtuins), ten-eleven translocation (TET) family proteins, and the C-terminal Binding Protein family [22]. Because the adenovirus genome is chromatinized through rapid association with cellular histones upon entry into the host cell nucleus, viral gene expression is likely regulated by these cellular chromatin-modifying mechanisms and responsive to immunoactivation of the host lymphocyte [23–25].

The C-terminal Binding Protein (CtBP) family of transcriptional corepressors was discovered through their high affinity binding to AdV E1A proteins [26, 27]. Mammalian cells express both CtBP1 and its homolog CtBP2 (collectively known as CtBP), which both share a 2D-hydroxyacid dehydrogenase domain, RRT-binding domain, and the PxDLS-binding domain responsible for the interaction with E1A (reviewed in [28]). CtBP homo- and hetero-dimers also likely form tetramers with the capacity to recruit many different chromatin modulators including Class I and II HDACs, histone methyltransferases, E3 ligases and other transcriptional regulators into large transcriptionally repressive complexes at the promoters of genes [28–31]. The assembly and stability of these complexes are dependent on nicotinamide adenine dinucleotide (NAD^+^ and its reduced form NADH) binding, and CtBP has been reported to function as an NAD(H) sensor and therefore a link between metabolic state and transcriptional regulation [30, 32–36].

Much has been reported about CtBP and its interaction with the viral E1A proteins. Initiation of the lytic AdV infection is marked by expression of the immediate early gene *E1A*, which has two main protein isoforms - large (13S E1A, 289R) and small (12S E1A, 243R) - responsible for transactivating other viral early genes and driving expression of cellular S-phase genes, respectively [37]. Both E1A isoforms interact with high affinity with both CtBP1 and CtBP2 through a PLDLS-motif located in the shared conserved region 4 (CR4) at the C-terminal end of the E1A proteins. Large E1A has an additional CtBP interaction domain located in the CR3 region unique to this isoform [38]. Of note, NADH was found to facilitate binding of CtBP to E1A at 1000-fold lower concentration than NAD^+^, suggesting that the NAD^+^/NADH ratio in the cell may affect the formation of CtBP-E1A protein complexes [32].

The role of the CtBP-E1A interaction in the lytic AdV life cycle is complex and has been reported to be either repressive or faciliatory, depending on the context. Mutation of the CtBP-binding site in CR4 of E1A drastically reduces virus replication, but stable knock-down of CtBP2 increases viral yield [39, 40]. CtBP1 and CtBP2 suppress the *ras*-cooperative transformative activity of the E1A proteins, but are required for E1B-55 K cooperative transformation [26, 39, 41–43]. At the level of transcriptional regulation, CtBP has been found to both repress and enhance E1A transactivation of viral and cellular genes [38, 44]. In a reciprocal relationship, E1A can exert influence over CtBP function as well, such as by altering acetylation and repressor-complex composition [44] and enhancing nuclear localization [45, 46]. These findings suggest that the high affinity binding between the E1A proteins and the CtBP proteins could form different context-specific complexes with finely-tuned functions. Given the complex nature of CtBP function during lytic infections of epithelial cells, it seems plausible that the CtBP proteins function in yet a different capacity within the unique cellular backdrop of persistent infection in lymphocytes.

The present study focuses on the mechanisms of viral reactivation in lymphocytes infected with AdV-C and provides experimental evidence for metabolically-linked mechanisms that could contribute to viral reactivation following cell activation. We show that viral transcription in lymphocyte models of AdV persistence is repressed compared to lytically-infected cells, but that relative amounts across viral transcripts are similar between the two infection types. Our data reveal that activation of lymphocytes shifts the NAD^+^/NADH ratio and that viral transcription is linked to alterations in this ratio. We also report differential expression of the NAD-dependent CtBP protein homologs between lymphocytes and epithelial cells. Last, our data reveal that inhibition of CtBP interaction with PxDLS-motif binding partners upregulates AdV *E1A* expression in T lymphocytes but not epithelial cells. Together, our results provide novel insight into metabolic factors that can regulate adenoviral reactivation in human lymphocytes.

## Material and methods

### Cell lines

The human lung carcinoma cell line A549 was purchased from the American Type Culture Collection (ATCC, Manassas, VA). BJAB (EBV-negative Burkitt’s lymphoma, [47]) and Jurkat (T cell Acute Lymphoblastic Leukemia [ALL]) were also obtained from the ATCC. KE37 (immature T cell ALL) cells were purchased from the German Collection of Microorganisms and Cell Cultures (DSMZ, Braunschweig, Germany). Me-180 (HPV-positive cervical carcinoma) and CaLu1 (lung carcinoma) were obtained from Linda R. Gooding (Emory University, Atlanta, GA). A549 cells were grown in Dulbecco’s modified Eagle medium (DMEM) with 4.5 μg of glucose per ml, 10% fetal calf serum (FCS), and 10 mM glutamine. BJAB, Jurkat, and KE37 cells were grown in RPMI medium supplemented with 10% FCS and 10 mM glutamine. Me-180 and CaLu1 were grown in McCoy’s medium, 10% FCS, and 10 mM glutamine. Cells were routinely evaluated to ensure the absence of mycoplasma and lymphocyte cell lines were authenticated by Genetica Cell Line Testing (Burlington, NC).

### Adenoviruses

The AdVC-5 mutant virus strain Ad5dl309 is phenotypically wild-type in cell culture and was obtained from Tom Shenk (Princeton University, Princeton, NJ). Ad5dl309 lacks genes necessary for evading adaptive immune attack (E3 RIDα and RIDβ proteins as well as the 14,700-molecular-weight protein (14.7 K protein)) in infected hosts [48].

### Infection of lymphocytes with adenovirus

Infection of lymphocyte cell lines with adenovirus was performed as described previously [49] with minor modifications. Lymphocytes were collected and washed in serum-free (SF) RPMI medium, and cell density was adjusted to 10^7^ cells per mL in SF-RPMI medium. Virus was added to the cell suspension at 50 PFU/cell, spun for 45 min at 1000 x g at 25 °C, and resuspended by agitation. Cells were then incubated at 37 °C for 1.5 h with gently flicking every 30 min. The infected cells were washed three times with complete RPMI medium and then resuspended in complete RPMI medium at 5 × 10^5^ cells per mL for culture. Cell concentration and viability were monitored throughout the infection. Replicates for experiments were obtained from independent infections.

### Stimulation of immune cell activation

Lymphocytes were treated for 24 h with 81 nM PMA + 1.35 μM Ionomycin (1X EZCell™ Cell Stimulation Cocktail, BioVision, Milpitas, CA). Following Fc block treatment (BD Pharmingen, San Jose, CA), cells were stained with fluorophore-conjugated antibodies against CD69 (PE, Biolegend, clone FN50) and CD25 (FITC, BioLegend, clone BC96), or stained with isotype control, and assessed by flow cytometry using LSR Fortessa (Becton Dickinson) and FlowJo Software (Becton Dickinson).

### Drug treatments

Drug treatment concentration and time of exposure were optimized for all cell lines. For lymphocytic and epithelial cell lines, cells were seeded at a density of 3 × 10^5^ and 1 × 10^5^ cells per mL, respectively, in complete medium supplemented with treatment doses of drugs. Treatment drugs and doses tested include nicotinamide (NAM, Sigma-Aldrich, [2, 5, 10 mM]) and NSC95397 (CtBP inhibitor, Tocris, Bristol, UK, [0.5, 1, 5, 10, 20 μM]). Cell growth and viability were assessed by Trypan blue exclusion at 12 (NSC95397 only), 24, and 48 h. Experiments utilized the following doses which maintained the viability indicated: NAM-10 mM, > 80% for 48 h; NSC95397–10 μM for 24 h, which maintained > 40% viability in lymphocytes and > 70% viability for epithelial cells.

### Reverse transcription and quantitative PCR analysis of viral and cellular mRNA levels

RT-qPCR was performed as described previously with minor modifications [50]. Briefly, total RNA was isolated from 1 × 10^6^ cells using the RNeasy Mini Kit (Qiagen Inc., Valencia, CA) with RNase-free DNase treatment (Qiagen). After spectrophotometric quantification, 200 ng of RNA was reverse transcribed into cDNA in 20 μL reactions (Maxima First Strand cDNA Synthesis Kit, Thermo Fisher Scientific, Waltham, MA). RT-enzyme negative controls were included for each reaction. Primers and probes were obtained from Integrated DNA Technologies (Coralville, IA), with sequences specified below. Each cDNA sample was run in duplicate qPCR reactions using the Maxima Probe/ROX qPCR Master Mix (Thermo Fisher Scientific) with cycling conditions as described.

For all experiments in which changes to viral gene transcription were assessed and expression of our housekeeping gene (eukaryotic translation initiation factor 1, [*EIF1*]) was unchanged by treatment, we quantified relative amounts of target (fold-change over untreated) as ${2}^{-\left(\Delta  {C}_{T, treated}-\Delta  {C}_{T, untreated}\right)}={2}^{-\Delta  \Delta  {C}_T}$ as described in [51]. In experiments using NSC95397, four different housekeeping genes (*GAPDH*, *HPRT1*, *ACTB*, and *EIF1*) were all negatively impacted by treatment. Because our primer amplification efficiencies are similar, and cDNA was prepared using equal amounts of RNA for all treatments, we used ${2}^{-\Delta  {C}_T^{\prime }}={2}^{-\left({C}_{T, treated}-{C}_{T, untreated}\right)}$ [51] for each gene separately, and present the down-regulated housekeeping gene for reference. This formula was also used for comparing relative amounts across different viral transcripts of untreated samples. We approximate the constant *K* = 1 (represents the ratio between the target gene and the housekeeping gene of the number of molecules present at threshold cycle given an initial number of molecules, defined in Eq. 4 [51]). For this, ${2}^{-\Delta  {C}_T^{\ast }}={2}^{-\left({C}_{T, target\ gene}-{C}_{T, housekeeping\ gene}\right)}$ was used to yield an approximate relative amount of target compared to the housekeeping gene for each viral gene.

Primers and Probes:

E1A (Sense sequence, 5′- GTTAGATTATGTGGAGCASCCC-3′, anti-sense sequence, 5′-CAGGCTCAGGTTCAGACAC − 3′, probe sequence, 5′-6 FAM-ATGAGGACCTGTGGCATGTTTGTCT-3IABkFQ-3′).

E3GP19K (Sense sequence, 5′-TTTACTCACCCTTGCGTCAG-3′, anti-sense sequence, 5′-GCAGCTTTTCATGTTCTGTGG-3′, probe sequence, 5′-6 FAM-CTGGCTCCTTAAAATCCACCTTTTGGG-3IABkFQ-3′).

TLP HEXON (Sense sequence, 5′-AAAGGCGTCTAACCAGTCAC-3′, anti-sense sequence, 5′-CCCGAGATGTGCATGTAAGAC-3′, probe sequence, 5′-6 FAM-CGCTTTCCAAGATGGCTACCCCT-3IABkFQ-3′).

EIF1 (Sense sequence, 5′- GATATAATCCTCAGTGCCAGCA-3′, anti-sense sequence, 5′-GTATCGTATGTCCGCTATCCAG-3′, probe sequence, 5′-6 FAM-CTCCACTCTTTCGACCCCTTTGCT-3IABkFQ-3′).

### Quantitative real time PCR analysis of viral DNA levels

Infected or uninfected control cells were washed in phosphate-buffered saline (PBS) and 5 × 10^5^ cells for each sample were lysed in 100 μL of NP-40–Tween buffer containing proteinase K, as described in [5]. Samples were tested by real-time PCR for a region of *hexon* gene that is conserved among species C adenovirus serotypes. Samples were run in duplicate for each independent experiment, with cycling conditions as described. Viral genome numbers were quantified by comparison to an Ad2 DNA standard curve and normalized relative to *GAPDH* expression to account for small differences in cell input [5].

### Immunoblots for protein detection

Protein lysates were prepared using RIPA buffer (Sigma-Aldrich) with protease/phosphatase inhibitors (Cell Signaling Technologies), and protein concentrations were quantified using a BCA protein assay (Thermo Scientific). 30μg of protein was separated by sodium dodecyl sulfate-polyacrylamide gel electrophoresis (SDS-PAGE) on 7.5 to 12% polyacrylamide gels (Mini-PROTEAN TGX gels, BioRad, Hercules, CA). Proteins were transferred onto nitrocellulose membranes (Thermo Scientific) overnight at 30 mV at 4 °C. Following confirmation of protein transfer with Ponceau S staining (Aqua Solutions, Deer Park, TX), membranes were blocked at room temperature (RT) with 5% bovine serum albumin (BSA) for 1 h, washed three times with Tris-Buffered-Saline with 1% Tween (TBST), and incubated with primary antibodies on a rocker overnight at 4 °C. Following three washes with TBST, membranes were incubated with secondary HRP-conjugated antibodies for 1 h at RT. Membranes were washed three times with TBST, the HyGLO HRP chemiluminescent reagent (Denville, Quebec, CA) used as substrate, and signal detected using x-ray film (MTC Bio). Primary antibodies include CtBP1 (mouse, 612,042, BD Transduction Lab, San Jose, CA), CtBP2 (mouse, 612,044, BD Transduction Lab), and β-actin (rabbit, D6A8, Cell Signaling, Danvers, MA). Secondary antibodies used were also from Cell Signaling: HRP-linked anti-rabbit IgG (7074) and HRP-linked anti-mouse IgG (7076S).

### Quantification of total cellular NAD^+^ and NADH concentrations

NAD^+^ and NADH concentrations were determined using the bioluminescent NAD/NADH-Glo Assay from Promega (Madison, WI). Cells were plated at a density of 1.5–3 × 10^4^ cells per well in 250 μL complete media on 96-well plates. For determining the effects of treatments on NAD^+^/NADH ratios, cells were left untreated or drugs added, and all cells were incubated for times specified in figures. Nanomolar concentrations of NAD^+^ and NADH were determined following manufacturer’s instructions by comparison to a standard curve consisting of dilutions of β-Nicotinamide adenine dinucleotide (N8285, Sigma).

### Statistical analysis

Experiments were repeated at least three times unless otherwise indicated. The experimental data were analyzed using a student’s t-test in GraphPad Prism software. *P*-values less than 0.05 were considered statistically significant. Independent infections of lymphocytes exhibit a high degree of variability in gene expression preventing the ability to average observations across infections, thus for some experiments we have shown the results of independent replicate experiments.

## Results

### Viral transcription in persistently-infected lymphocytes is repressed compared to lytically-infected cells but relative amounts across viral transcripts are similar

**Fig. 1:**
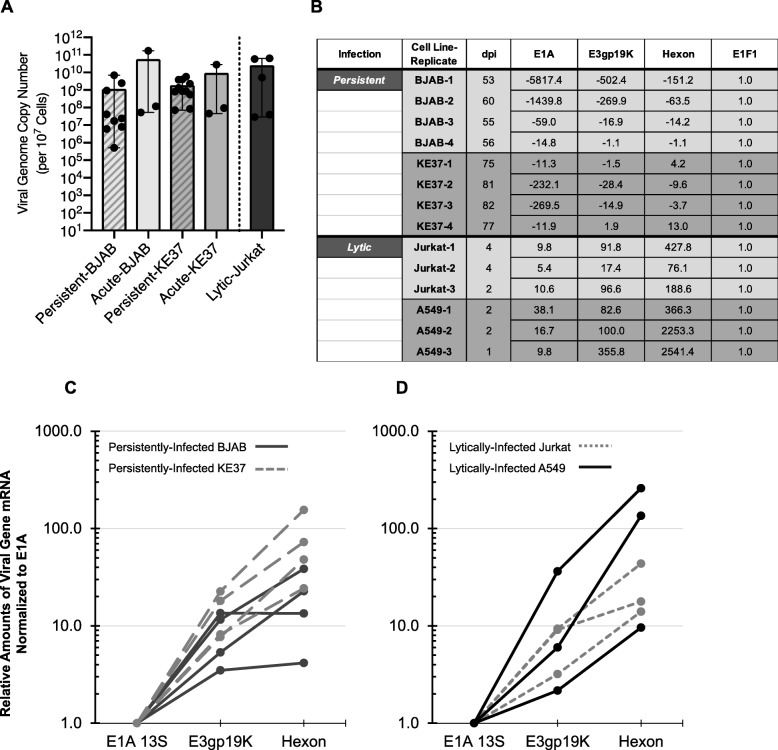
Characterization of viral genome quantities and transcriptional repression in persistently-infected lymphocytes. **a**) Viral genome copy numbers determined by qPCR as previously described (5). BJAB, KE37, and Jurkat were infected with a MOI 50. Error bars show median value with range. Cell information: BJAB, persistently-infected (*n* = 9) and acutely-infected (*n* = 3); KE37, persistently-infected (*n* = 9) and acutely-infected (*n* = 3); lytically-infected Jurkat (*n* = 5). Persistently-infected lymphocytes were evaluated between 50 to 241 dpi, lytically-infected Jurkat cells were evaluated at 2 to 4 dpi. **b**) Viral transcription in persistently-infected lymphocytes was determined by RT-qPCR and relative amounts of mRNA calculated as described in *Materials and Methods* and normalized to the housekeeping gene *EIF1* (which was not affected by infection, and was set to 1). The negative reciprocal was taken for values less than one to show down-regulation on the same scale. **c** & **d**) Relative amounts of viral transcripts E1A-13S, E3gp19K, and hexon in persistently-infected (**c**) and lytically-infected cells (**d**) were determined by RT-qPCR using equal amounts of RNA. Relative amounts were calculated as described in *Material and Methods* and then normalized to E1A (which was set to 1). Experiments were repeated at least 3 times with similar results. BJAB (*n* = 4, 53–60 dpi), KE37 (*n* = 4, 5–82 dpi), Jurkat (*n* = 3, 2–4 dpi), A549 (*n* = 3, 1–2 dpi)

Lymphocytic cell line models of infection harbor high levels of viral DNA for long periods of time, with very low amounts of detectable viral proteins [17, 21]. As these cell-line infections progress over time, viral genome levels decline from peak levels during the “acute phase” (1–30 days post infection (dpi)) into the “persistent phase” (> 30 dpi). The viral genome is retained during persistence for more than 100 dpi at 10–1000 copies per cell [17, 18]. To further characterize the persistent phase dynamics, we examined the variability in the viral load across several independent infections. Using qPCR, we quantified viral genome copy number during both the acute and persistent phases of two persistently-infected lymphocytic cell lines (BJAB and KE37) and compared those to acutely-infected lymphocytes as well as lytically-infected cells (Jurkat) (Fig. 1a). Acutely-infected BJAB and KE37 were found to carry similar viral loads to lytically-infected Jurkat cells (1 × 10^8^–1 × 10^11^ copies per 10^7^ cells). These levels are similar to those previously detected in lytically-infected epithelial cells (1.2 × 10^11^–1.6 × 10^11^ copies per 10^7^ cells 48 h post-infection with MOI 30) [49]. On average, persistently-infected cells harbor fewer copies of the viral genome than acutely-infected counterparts, though the differences are not significant (Fig. 1a). Notably, lymphocyte infections are capable of maintaining 2 to 4-log differences in quantities of viral DNA infection-to-infection (1 × 10^5^–1 × 10^9^ copies per 10^7^ cells). This variability in viral genome copy number has also been reported for naturally-infected mucosal lymphocytes which can range from 1 × 10^2^ to 1 × 10^7^ copies per 10^7^ cells [2, 8].

We have previously reported that expression of the adenovirus death protein (ADP) is repressed in persistently-infected lymphocyte cell lines [21]. Krzywkowski et al. (2017) also showed reduced E1A and MLP mRNA levels in persistently-infected BJAB cells, relative to lytically-infected HeLa cells even when viral DNA levels were comparably high [19]. To extend these observations to other viral genes we quantified transcription from three genes expressed during immediate early (*E1A*), early (*E3*), and late (*hexon*) adenovirus infection. Quantities of viral transcripts from persistently-infected BJAB and KE37 cells were determined relative to a cellular housekeeping gene *EIF1* (which was not altered by infection, data not shown). We compared persistent quantities to viral transcripts in lytically-infected Jurkat and A549 cells. In lytically-infected cells, all viral transcripts were expressed at levels higher than the cellular reference gene (Fig. 1b). Interestingly, viral transcription was markedly lower in lytically-infected Jurkat compared to A549, which may contribute to the delayed lysis reported for this infection [17]. As expected, persistently-infected cells showed severely repressed levels of viral transcripts compared to lytically-infected cells, suggesting that for a substantial proportion of viral genomes infecting these cells, transcription is repressed.

While viral gene expression was repressed in persistent infection, we sought to determine if viral expression of these same three genes (*E1A*, *E3*, and *hexon*) was maintained at expected amounts relative to one another. During the course of lytic infections in epithelial cells, the viral gene expression program follows a well-described progression [52–54]. When maximum rates of transcription are evaluated, E1A mRNA is present in infected cells in lower amounts than that of E3. Hexon mRNA and other late mRNA quantities are much larger than those of early genes [54–56]. To directly determine if viral transcript ratios seen in lytic infection were similar in persistent infection, we quantified relative viral transcription in persistently-infected BJAB and KE37 cells and compared them to relative transcript amounts in lytically-infected A549 and Jurkat cells. The fold change of both E3gp19K and hexon mRNA relative to E1A mRNA levels are shown in Fig. 1c and d. On average, E3 was 10-fold greater than E1A while hexon was 30-fold greater than E1A. Moreover, despite the variability in genome copy number across samples (Fig. 1a), relative quantities of E1A, E3gp19K, and hexon mRNA in persistently-infected cells (Fig. 1c) are not distinguishably different from ratios in lytically-infected cells (Fig. 1d), indicating that persistently-infected cells expressing these genes are producing them at expected ratios.

### Cellular activation of infected lymphocyte cell lines upregulates viral gene expression

**Fig. 2:**
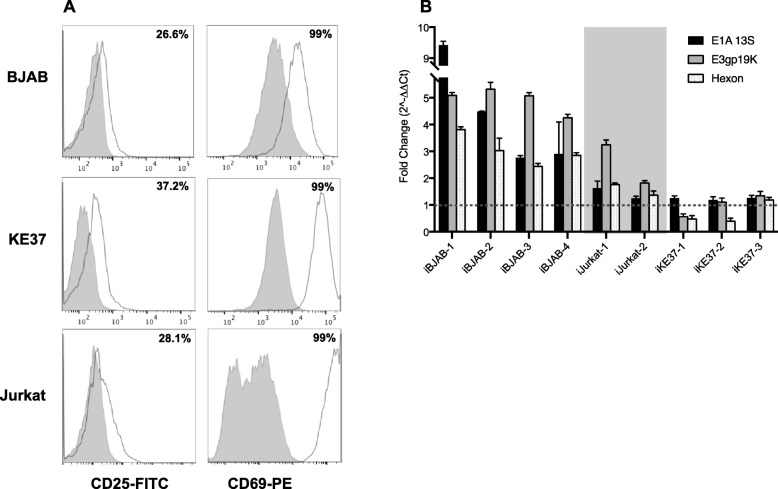
Cell stimulation with PMA and Ionomycin upregulates viral gene expression in infected lymphocytic cell lines. **a**) Infected BJAB, KE37, and Jurkat cells were stimulated with PMA/Iono for 24 h and stained with monoclonal antibodies for CD25 (FITC-labeled) and CD69 (PE-labeled) detected by flow cytometry. Percentages indicate number of cells positive for the indicated marker following stimulation. Shaded area and open area show untreated and PMA/Iono-treated samples, respectively. **b**) PMA/Iono-induced changes to viral gene expression were evaluated in persistently-infected BJAB or KE37 cells (between 50 to 100 dpi) and lytically-infected Jurkat cells (2 dpi). Cells were treated for 24 h with PMA/Iono and changes to viral gene expression assessed by RT-qPCR. Lytically-infected Jurkat are demarked by the shaded region to differentiate from persistently-infected cells. Four infected BJAB, three infected KE37, and two infected Jurkat replicate infections are shown. Fold change is shown over untreated samples (set to 1). Dashed gray line shows the line of *fold change* = 1. Error bars show standard deviation in replicate wells

Immune cell activation with a cocktail of activating agents (PMA, Ionomycin, IL-2, anti-CD3 and anti-CD28) has previously been shown to reactivate viral transcription and induce production of infectious particles in latently-infected tonsillar lymphocytes [2]. To determine if our infected cell line models would respond similarly, we first confirmed that immune cell signaling in our lymphocytic cell lines was functional. Cells were activated with PMA/Iono for 24 h and the surface expression of CD25 and CD69, markers of lymphocyte activation, was measured by flow cytometry [57]. Stimulation induced upregulation of both CD25 and CD69 compared to basal levels in all three cell lines (Fig. 2a). We next evaluated viral *E1A*, *E3*, and *hexon* expression levels after cell activation. Stimulation upregulated viral gene expression in all three lymphocyte lines compared to untreated cells. Upregulation was most robust in the BJAB cells (~ 4-fold, 5-fold, and 3-fold for *E1A*, *E3*, and *hexon*, respectively) and small but detectable in *E1A* in the KE37 cells (1.2-fold average increase, Fig. 2b). Of note, *E1A* responded in all 3 replicates of infected KE37 while *E3* was increased in 2 of 3 experiments. Overall, the viral early genes were more responsive to stimulation with PMA/Iono than the late gene *hexon*. In this regard, a PMA-responsive element has previously been reported in the *E1A* promoter [58]. Further, PMA has been reported to act synergistically with E1A protein to upregulate *E3* expression [59]. Thus, these two actions of PMA at these early genes may contribute to the increases in viral early gene expression detected here in response to stimulation. Interestingly, PMA/Iono was also able to upregulate viral early gene expression in lytically-infected Jurkat cells at a level intermediate between the persistently infected BJAB and KE37 cell lines.

### Infection with adenovirus can reduce the NAD^+^/NADH ratio and PMA/ionomycin stimulation shifts this ratio in lymphocytic cell lines

**Fig. 3:**
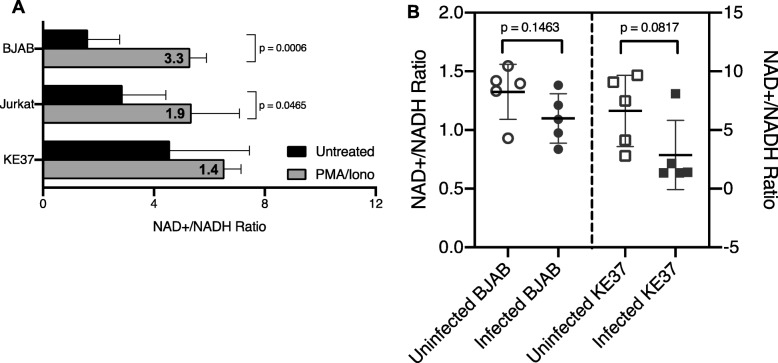
PMA and ionomycin treatment increase NAD^+^/NADH ratio in lymphocyte cell lines. **a**) Uninfected cells were treated with PMA/Iono for 4 h, and total cellular NAD^+^ and NADH nM concentrations were determined using a bioluminescent assay with standard curve. Numbers of replicates: BJAB - untreated, *n* = 8, treated, *n* = 3; Jurkat – untreated, *n* = 8, treated, *n* = 3; KE37 – untreated, *n* = 5, treated, *n* = 3. Fold-increase in treated over untreated is shown in bold in the gray bar (mean of the treated/mean of the untreated). Error bars show standard deviations of the NAD^+^/NADH ratios. *P*-values were determined using a student’s t-test. **b**) Impact of persistent infection on NAD^+^/NADH ratios. Total nM NAD^+^ and NADH were determined in persistently infected lymphocytes (> 50 dpi) as described in *Material and Methods*. For all samples, *n* = 5. Mean and standard deviation of the ratios is shown. P-values were calculated using student’s t-test

Lymphocytes remain in a resting state until activated and can undergo dramatic shifts in transcriptional programs upon activation [60–62], as well as shifts in metabolism resulting in significant increases in NAD^+^ and NADH concentrations [63]. These changes can impact transcription via chromatin remodelers dependent upon specific concentrations of metabolites as co-substrates or co-factors [64]. To begin to understand some of the cellular mechanisms behind the PMA/Iono-induced upregulation of viral gene expression in infected lymphocytes, we first measured the impact of PMA/Iono stimulation upon cellular NAD^+^/NADH ratios in our lymphocytic cell lines. Treatment with PMA/Iono increased the NAD^+^/NADH ratio in our three lymphocyte cell lines, with a significant 3.3-fold increase in BJAB (*P* = 0.0006) and a 1.9-fold increase in Jurkat (*P* = 0.0465) (Fig. 3a). KE37 had the highest average NAD^+^/NADH ratio when untreated. This cell line also had the widest range of NAD^+^/NADH-ratio values in an unstimulated state, and though we observed an increase in ratio for KE37 after PMA/Iono treatment, it was not statistically significant. This cell line also exhibited the smallest increase in viral gene expression by PMA/Iono (Fig. 2b).

In the course of lytic infection of epithelial cells, AdV is known to alter metabolic pathways of the host cell, such as glycolysis and the tricarboxylic acid (TCA) cycle, to generate the metabolites and macromolecular precursors demanded by viral replication (reviewed in [65]). Whether persistent adenovirus infection results in metabolic reprogramming of the host cell is not known, although persistently-infected cells continue to divide normally as one measure of cellular activity [17]. If viral gene expression is linked to the NAD^+^/NADH ratio of the cell, and treatments which increase the NAD^+^/NADH ratio increase viral gene expression (Fig. 2 & 3b & a), we wondered if the NAD^+^/NADH ratio was reduced in persistently-infected cells where viral gene expression is repressed. To address this question, we measured the NAD^+^/NADH ratio in persistently-infected BJAB and KE37 cells compared with their uninfected counterparts (Fig. 3b). On average, the NAD^+^/NADH ratio is reduced in persistently-infected lymphocytes compared to uninfected controls and approaches significance in KE37 cells (*P* = 0.0817). BJAB cells, however, have a much lower baseline ratio as compared to KE37 (1.4 vs 6, respectively), and infection appears to moderately reduce it further, though not to statistically significantly levels.

### Direct modulation of the NAD^+^/NADH ratio can upregulate viral gene expression in persistently-infected cells

**Fig. 4:**
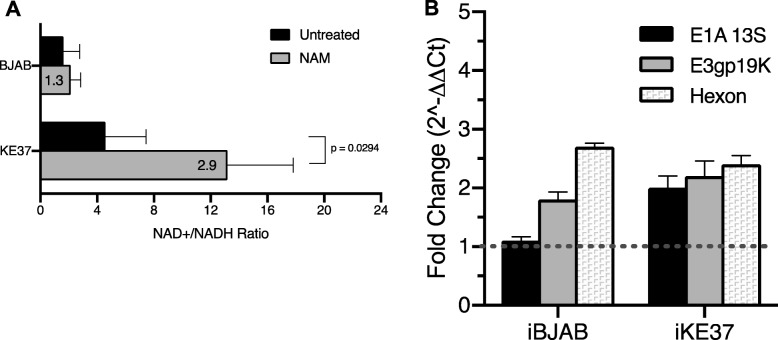
Viral gene expression is responsive to the NAD^+^/NADH ratio. **a**) Uninfected cells were treated with 10 mM NAM for 24 h, and total cellular NAD^+^ and NADH concentrations determined as described previously. *N* = 3 for all cell lines. Fold-increase in treated over untreated is shown in bold in the gray bar (mean of the treated/mean of the untreated). Error bars show standard deviations of the NAD^+^/NADH ratios. P-values were determined using a student’s t-test. **b**) Impact of NAM on viral gene expression in persistently-infected cells. Persistently-infected BJAB and KE37 (> 50 dpi) were treated with 10 mM NAM for 24 h. Following RT-qPCR, fold-change in viral is calculated using 2^−∆∆*Ct*^ as described in the *Material and Methods*, with untreated sample as reference (set to 1) and cellular gene *EIF1* as the housekeeping gene. Dashed gray line shows *fold change* = 1. Error bars show standard deviation in replicate wells. One representative experiment for each cell line is shown. This experiment was repeated three times with similar results using BJAB cells. This experiment was repeated four times for hexon in KE37 cells with similar results and twice for E1A and E3 with similar results

To more directly evaluate the impact that shifts in the NAD^+^/NADH ratio could have on viral gene expression, we treated cells with nicotinamide (NAM) which has been reported to increase the NAD^+^/NADH ratio [66]. As expected, NAM treatment increased the NAD^+^/NADH ratio in BJAB (1.3 fold) and more significantly altered KE37 (2.9-fold, *P* = 0.0294). Again, Jurkat fell in between these 2 cell lines with a 1.9-fold increase (*P* = 0.0706, data not shown). Following NAM treatment of persistently-infected lymphocytes, we measured the impact of increasing the NAD^+^/NADH on viral gene expression. As shown in Fig. 4b, treatment with NAM increased viral gene expression of early and late genes in both persistently-infected cell lines. *E1A* and *E3* expression appeared to be more robustly increased in KE37 as compared to infected BJAB cells. Moreover, these NAM-induced increases in viral gene transcription could be seen at the protein level by flow cytometry during the acute phase of infection when viral proteins are expressed at detectable levels, and both BJAB cells and KE37 cells exhibited increased expression of hexon protein following treatment with NAM at 20 dpi (data not shown). Interestingly, the increases in viral gene expression detected, following treatment with either PMA/Iono and NAM, appear to correspond to the increases detected in NAD^+^/NADH ratio. In KE37, NAM shifted the NAD^+^/NADH ratio 2.9-fold (Fig. 4a) compared to 1.4-fold with PMA/Iono (Fig. 3a). NAM similarly increased viral mRNA more robustly (> 2-fold for all 3 viral genes) (Fig. 4b) than did PMA/Iono treatment (< 1.5-fold for *E1A* only) (Fig. 2b). In BJAB cells, PMA/Iono induced a larger shift in the NAD^+^/NADH ratio than did NAM (3.3-fold compared to 1.3-fold, respectively). PMA/Iono also induced larger increases in viral gene expression (Fig. 2b) than NAM (Fig. 4b) (> 3-fold compared to < 3-fold). These results suggest that viral gene expression in lymphocytes could be tied to the NAD^+^/NADH ratio of the host cell.

### Differential expression of CtBP homologs between lymphocytes and epithelial cells

**Fig. 5:**
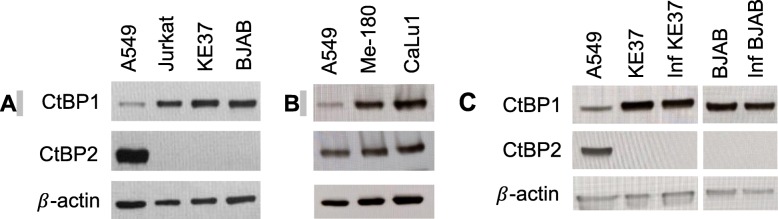
Epithelial cells and lymphocytic cells differ in CtBP2 expression. Western blot analysis of CtBP1 and CtBP2 proteins was performed on epithelial and lymphocytic cell as described in the *Material and Methods* (**a**, **b**, **c**). Permissive epithelial cells lines A549 (lung), Me-180 (cervical) and CaLu-1 (lung) are shown. β-actin protein levels were used as a control for equal protein loading. **c**) Persistently-infected KE37 and BJAB at 121 and 101 dpi, respectively

The AdV genome remains episomal in lymphocytes [17] and associates with cellular histones in infected cells [24, 25, 33]. CtBP repressor complexes associate with histones to regulate gene expression and are sensitive to NAD^+^/NADH levels [35]. Moreover, these proteins were discovered more than two decades ago through their high affinity interactions with AdV E1A proteins (289R and 243R, large and small E1A respectively) [26, 27]. E1A large and small proteins are the first to be expressed upon infection and are critical for auto-activating the *E1A* gene, transactivating expression of other early viral genes, and driving the cell into S-phase [67]. Thus, these proteins must be tightly controlled in cells where persistence, and not lysis, is the outcome. CtBP has paradoxically been reported to both repress and potentiate AdV infections during lytic infection of epithelial cells [26, 38, 39, 41–44]. We thus wanted to investigate if the CtBP proteins could be involved in the repression of viral transcription during persistent infection in lymphocytes. Although CtBP1 and CtBP2 share a high degree of homology, differences in expression patterns, structure, and localization suggest context-dependent functions of these co-repressors. To begin understanding if these proteins could be contributing to AdV gene repression we first evaluated the CtBP protein levels in our cells and discovered striking differences between lymphocytic and epithelial cell lines. We found that CtBP2 was undetectable in all lymphocyte cell lines compared to the lung epithelial cell line A549 (Fig. 5a). To determine if the high level of CtBP2 expression was a characteristic of other AdV-permissive epithelial cell lines, we evaluated two additional epithelial cell lines, Me-180 (cervical) and CaLu-1 (lung) [68, 69] (Fig. 5b). We detected similarly abundant amounts of CtBP2 in these epithelial cells. CtBP1 expression was consistent across the cell lines, with the exception of A549 cells which had the lowest amount of CtBP1 protein among all the cell lines. Because persistent infection has been shown to alter expression of some cellular proteins in lymphocytes [17], we confirmed that CtBP1 was expressed at similar levels in both uninfected and persistently-infected lymphocytic cell lines (Fig. 5c). Persistent infection also did not alter CtBP2 protein levels in lymphocytes, which remained undetectable (Fig. 5c). The striking difference in the CtBP expression profiles between epithelial cells and lymphocytes suggests that CtBP could be impacting adenovirus infection differently in lymphocytes as compared to what has been previously reported in epithelial cells [44–46].

### Inhibition of CtBP-E1A interaction upregulates E1A 13S expression in T lymphocyte cell lines

**Fig. 6:**
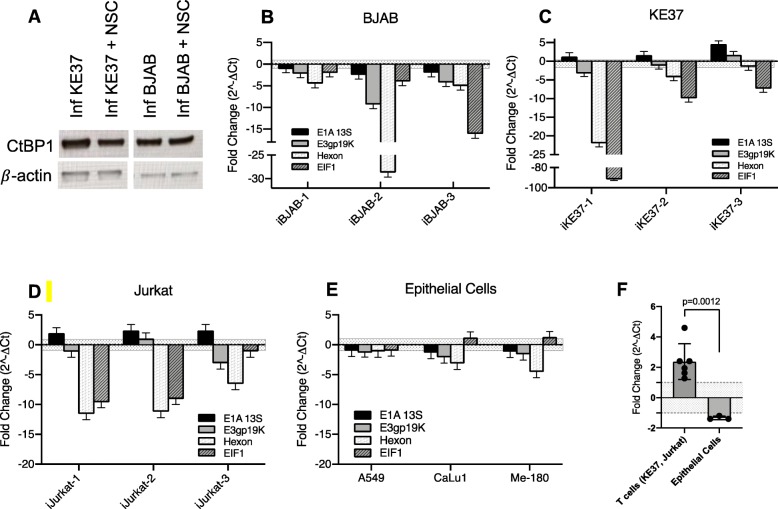
CtBP binding inhibitor, NSC95397, differentially impacts AdV gene expression across lymphocytic and epithelial cell lines. **a**) Western blot analysis of CtBP1 protein was performed as described in *Materials and Methods* on cell lysates collected with or without 24-h treatment with NSC95397. Both persistently-infected KE37 and BJAB were 66 dpi. **b**-**e**) RT-qPCR and analysis to assess viral gene expression performed as described for NSC95397 treatment in *Material and Methods*. **b**) Persistently-infected BJAB (≥ 61 dpi, *n* = 3), **c**) persistently-infected KE37 (≥ 80 dpi, *n* = 3), **d**) lytically-infected Jurkat (2 dpi, *n* = 3). Down-regulated values are shown as the negative reciprocal, which eliminates values falling between − 1 and 1 (indicated by shaded zone). Error bars show standard deviation on replicate wells. **e**) Lytically-infected epithelial cells A549 (2 dpi, error bars represent the SD of 3 independent experiments), CaLu1 (2 dpi, *n* = 1, SD of replicate wells), and Me-180 (2 dpi, *n* = 1, SD of replicate wells). **f**) Comparison of average change in *E1A* expression induced by NSC95397 treatment in T cell lines (KE37 and Jurkat, data shown in Fig. 6c and d) and epithelial cells (data shown in Fig. 6e). Shaded zone between − 1 and 1 as described above. Error bars show SD of fold-change values. P-value was determined using student’s t-test

To examine the role CtBP might have on viral transcription in lymphocytes, we utilized the small molecule inhibitor NSC95397. This compound specifically blocks binding between CtBP and PxDLS-containing partners and has been shown to disrupt the CtBP1-E1A interaction [70]. First, we confirmed that treatment with NSC95397 did not alter CtBP1 protein levels in persistently-infected lymphocytes (Fig. 6a), and CtBP2 likewise remained undetectable (data not shown). We next examined the effect of NSC95397 treatment on viral gene expression in persistently-infected lymphocytic cell lines. Treatment of BJAB cells with NSC95397 caused down-regulation of all viral genes across three independent experiments (Fig. 6b), however, *E1A* expression was the least impacted. E1A mRNA decreased 1.5- to 3-fold compared to the larger decrease in hexon (4- to 30-fold). Surprisingly, NSC95397 induced a more robust down-regulation of the cellular housekeeping gene *EIF1* (2-, 4- and 16-fold). We tested 3 additional housekeeping genes (glyceraldehyde-3-phosphate dehydrogenase [*GAPDH*], hypoxanthine phosphoribosyltransferase 1 [*HPRT1*], and β-actin [*ACTB*]) across all lymphocyte lines and saw robust down-regulation of each of them ranging from 2- to 11-fold (data not shown). Interestingly, the down-regulation of the housekeeping gene in BJAB cells was greater than the down-regulation observed for *E1A*. Because of the robust down-regulation of multiple housekeeping genes tested in our study, fold-changes in gene expression between treated and untreated cells are shown without normalization to an endogenous control as described in *Material and Methods* [51].

Inhibition of CtBP binding with PxDLS-containing partners using NSC95397 also caused decreases in hexon mRNA in both KE37 cells (2- to 20-fold) and Jurkat cells (5- to 10-fold) (Fig. 6 c & d). CtBP inhibition, however, has a noticeably different effect on *E1A* expression in both of these T cell lines where *E1A* is upregulated by 1.5- to 4-fold. The expression of *E3* was minimally impacted in these cells. These data suggest that CtBP binding with PxDLS-containing partners may be repressing transcription of *E1A* in T cells and that inhibiting this binding allows for expression. In contrast, CtBP may paradoxically be necessary for expression of the viral late gene *hexon* in lymphocytes*,* since it was maximally downregulated by NSC95397 treatment in both the B and T cell lines.

All of the lymphocyte cell lines have delayed infection dynamics as compared to infected epithelial cells [49]. In addition, though Jurkat cells undergo a lytic infection with AdV-C5, they still exhibit much reduced levels of viral gene expression (Fig. 1b). To find out if inhibiting CtBP binding with PxDLS-containing partners would have the same effect on viral transcription in epithelial cells, we initiated treatment with NSC95397 in lytically-infected epithelial cells. As shown in Fig. 6e, NSC95397 treatment had almost no impact on viral gene expression in A549 cells. Because the lytic life cycle in A549 is rapid and usually complete by 48 h, we also assessed viral gene expression at 6 h post-infection (5 h after the addition of NSC95397). No effect of NSC95397 treatment could be seen at this earlier time point in infection (data not shown). Interestingly, when we assessed viral transcription in two other epithelial cell lines, CaLu1 and Me-180, NSC95397 treatment negatively impacted *hexon* expression, though not nearly to the level observed in lymphocytes, causing 3- to 4-fold down-regulation (Fig. 6e). As with A549 cells, NSC95397 treatment did not induce any upregulation of *E1A* in these cells, and there was a negligible impact on the expression of the housekeeping gene. The significant difference in impact of NSC95397 treatment on *E1A* expression between T cell lines and epithelial cell lines (*P* = 0.0012) is shown in Fig. 6f. Overall, NSC95397 treatment strongly impacted both cellular and viral gene expression in infected lymphocytes (both persistently- and lytically-infected) but had much less impact on infected epithelial cells. Further, the unique gene expression changes do not appear to be wholly related to the cell sensitivity to NSC95397 toxicity as Me-180 cells displayed sensitivity similar to the lymphocytic cell lines (data not shown).

## Discussion

Most of what is known about adenovirus is from studies of lytically-infected cells, and much about adenovirus latency and reactivation is not well characterized. The virus can be life-threatening for immunocompromised individuals as well as pediatric transplant patients, however, the mechanisms that allow the virus to persist, or those that induce reactivation, are almost entirely unknown. Patient samples have shown that lymphocytes of the tonsils, adenoids [5], and gastrointestinal tract [8] contain AdV DNA and are presumably the sites of reactivation. The lack of small-animal models of persistent adenovirus infection has been an obstacle to studying infection dynamics in vivo, but a study of AdV infection using humanized mice has recently shown that persistently-infected cells could also be found in the bone marrow [71].

Our previous studies of AdV-infected lymphocytes from tonsils or adenoids suggest that replicating virus is more common among younger donors, however high genome copy number did not appear to correlate with active replication [2]. Replicating virus could be detected from cells containing a range of genome copy numbers, from as few as 10^4^ to as many as 10^6^ AdV genomes per 10^7^ cells [2]. Our cell line models of persistent lymphocyte infection carry AdV DNA levels in a range between 1 × 10^5^–1 × 10^9^ copies per 10^7^ cells (Fig. 1a). Within these persistently-infected models, many viral transcripts can be detected in low amounts with fewer than 1% of the cells expressing detectable levels of viral proteins or producing virus [20, 21].

The persistent phase of infection has been shown to be regulated, in part, by transcriptional controls not seen in lytic infections. Murali et al. (2014) determined that the *E3*-Adenovirus Death Protein (*ADP*) gene is repressed both transcriptionally and post-transcriptionally in cells which harbor persistent AdV infection [21]. Krzywkowski et al. (2017) showed that in persistently-infected BJAB, very few individual cells express E1A mRNA or Major Late Transcription Unit mRNA at levels comparable to lytically-infected HeLa cells, even when the cells harbored large amounts of viral DNA [19]. In contrast, Furuse et al. (2013) determined that persistently-infected BJAB expressed amounts of VA RNAI and VA RNAII that were comparable to those expressed in lytic infections. However, the relative proportion of the two transcripts differed when compared to lytic infection [20]. In our current study, we report low expression of both early (*E1A* and *E3*) and late genes (*hexon*) in infected lymphocytes as compared to lytically-infected cells (Fig. 1b). Indeed, the level of viral transcripts are all relatively lower than the expression level of the representative housekeeping gene. In contrast, AdV transcript levels are relatively higher than housekeeping gene expression in both the lytically-infected T cells (Jurkat) and lytically-infected epithelial cells (A549). However, we found reduced levels of viral transcripts in lytically-infected T cells as compared to lytically-infected epithelial cells revealing that lymphocytes in general have lower levels of AdV gene expression. We attempted to confirm differences in viral gene expression at the protein level but were unable to detect viral proteins which are in low abundance during viral persistence (data not shown). Despite some degree of transcriptional repression in the lymphocytes, viral mRNA ratios were surprisingly similar between persistently-infected and lytically-infected cells (Fig. 1c and d, respectively). These findings in lymphocytes are in line with amounts of E1A, E3, and hexon mRNAs (~ 4, 35, and 90%, respectively), quantified as a percent of GAPDH, at 36 h post-infection in normal lung fibroblasts recently reported by Crisostomo et al. (2019) [54].

Immunoactivation of tonsillar lymphocytes has been shown to reactivate latent AdV causing increases in viral gene expression and productive infection [2]. In previous studies, a cocktail of immune cell stimulators was used including PMA, Ionomycin, IL-2, anti-CD3 and anti-CD28, however, no specific mechanisms for viral gene de-repression were determined. In addition, these prior studies on activation of naturally infected lymphocytes were done using samples that contained both T cells and B cells together. In the current study, we report that PMA/Iono alone is sufficient to induce AdV gene expression in B and T cell models of persistent infection, as well as in lytically-infected Jurkat cells (Fig. 2b). In addition, we found that the magnitude of change in viral expression mirrors the change observed in the NAD^+^/NADH ratio (Fig. 3a). PMA/Iono treatment increased total cellular NAD^+^ and NADH concentrations (data not shown) and significantly increased the NAD^+^/NADH ratio in BJAB and Jurkat cells; large increases in AdV early gene expression were readily observable in these cells by 24 h. Stimulation, including PMA/Iono treatment, of resting lymphocytes has been well-documented to shift the metabolic program from primarily oxidative phosphorylation to glycolysis, which increases lactate production, increases synthesis of biosynthetic intermediates, and shifts the NAD^+^/NADH ratio [63, 72, 73]. Thus, our data support the notion that changes in the metabolic status of lymphocytes can promote reactivation of AdV gene expression. In the current study, PMA/Iono had the least impact on AdV gene expression in KE37 cells which corresponded with the non-significant change detected in the NAD^+^/NADH ratio in these cells. Whether the addition of other T cell stimulating agents (IL-2, anti-CD3 and anti-CD28) can induce a significant change in this ratio, as well as more robust changes in AdV gene expression, is still under investigation.

Interestingly, when comparing the basal NAD^+^/NADH ratios in the two persistently-infected cell lines, KE37 and BJAB, a trend toward viral infection reducing the NAD^+^/NADH ratio relative to their uninfected counterparts could be seen, though significance was not reached (Fig. 3b). These samples were evaluated at different times post-infection, and it is intriguing to speculate that AdV may significantly impact the NAD^+^/NADH ratio of the cells it persistently infects at some point during the course of the infection. How the virus would modulate cell metabolism mechanistically is unclear. Persistent adenovirus infection of B-lymphocytes has been shown to significantly down-regulate several cellular genes (*BBS9*, *BNIP3*, *BTG3*, *CXADR*, *SLFN11*, and *SPARCL* - [50]), however, none are reported to obviously function in the regulation of metabolism. Nonetheless, it is possible that some of the other genes identified as altered by AdV infection could play a role in this effect ([50], supplemental data).

Nicotinamide (NAM), which is recycled by the cellular NAD^+^-salvage pathway and converted into NAD^+^, can be used to manipulate the NAD^+^/NADH ratio of cells [74]. NAM treatment of persistently-infected cell lines significantly increased the NAD^+^/NADH ratio in KE37 while a much smaller change was induced in BJAB cells (Fig. 4a). Nonetheless, increased viral gene expression could be detected in both cell lines (Fig. 4b) suggesting that alterations in this metabolic ratio can induce viral gene expression in lymphocytes. Interestingly, in contrast to the robust PMA/Iono-induced upregulation of *E1A* and large increase in NAD^+^/NADH ratio seen in BJAB (3.3-fold, Fig. 2b), there was no apparent change in *E1A* expression when the ratio was only increased 1.3-fold with NAM (Fig. 4b). A similar relationship is seen between *E1A* expression and the shift in the metabolic ratio in KE37, where more *E1A* expression is seen following larger increases in the NAD^+^/NADH ratio (Figs. 4, 2b). These findings support a link between metabolic shifts in lymphocytes and the magnitude of AdV de-repression induced.

The link between the metabolic state of cells and gene expression contributes to lymphocyte functional responses following immune stimulation [64, 75, 76]. This transcriptional regulation involves chromatin remodelers dependent upon specific concentrations of metabolites that serve as co-substrates or co-factors [64]. CtBP is well-known repressor of gene expression that was discovered through its interaction with E1A [26, 27, 77]. CtBP tetramers associate with epigenetic enzymes forming complexes that modify the chromatin environment through coordinated histone modifications, allowing for the effective repression of genes targeted by DNA binding proteins associated with the complex [30–36, 78–80]. The stability of CtBP tetramers is dependent upon NAD(H) binding. Because AdV gene expression in lymphocytes is responsive to shifts in the NAD^+^/NADH ratio, we investigated whether CtBP, as a reported metabolic sensor, could be contributing to the transcriptional repression evident in persistent infection. When comparing CtBP protein levels, we found that our three lymphocyte cell lines only expressed CtBP1 and that CtBP2 protein could not be detected (Fig. 5a). CtBP2 expression has previously been reported to be in low abundance or undetectable in leukocytes, immune tissues, and lymphocyte cell lines [29]. In contrast to the lymphocytes evaluated in our study, A549 cells expressed high levels of CtBP2 with lower levels of CtBP1 (Fig. 5b). This finding suggested that the composition of CtBP complexes in lymphocytes is different than in epithelial cells, and therefore CtBP may interact differently with viral proteins in lymphocytes than what has been reported for epithelial cells.

NSC95397 is a small-molecule inhibitor of CtBP which acts through the disruption of CtBP binding to PxDLS-containing partners, including E1A [70]. Interestingly, treatment with NSC95397 resulted in mixed changes in expression of AdV genes (Fig. 6b-e). *E1A* expression was increased in the T cells lines (KE37 and Jurkat) but minimally impacted in the B cell line (BJAB). In sharp contrast to *E1A*, *hexon* expression was consistently downregulated across all the lymphocyte cell lines. The ability of NSC95397 to impact *E1A* expression in both a lytically-infected T cell line as well as a persistently-infected T cell line could indicate a T lymphocyte specific role for the disrupted interaction. Unlike the impact seen in T lymphocytes, none of the epithelial cell lines showed an increase in *E1A* expression with NSC95397 treatment (Fig. 6f). Among the epithelial cell lines, A549 showed negligible changes in AdV expression following treatment with NSC95397 while Me-180 and CaLu exhibited moderate downregulation of both *hexon* and *E3* (Fig. 6e). Whether this downregulation is attributable to the higher amount of CtBP1 present in these two epithelial cell lines as compared to A549 (Fig. 5b) is still unclear.

Of note, cell viability, especially that of transformed cell lines, can be negatively impacted following treatment with NSC95397 [70]. In our experiments, we optimized treatment timing to maintain cell viability at or above roughly 50% (data not shown). NSC95397 also induced substantial downregulation of multiple housekeeping genes (Fig. 6b-d, and unpublished data), although this effect did not directly relate to the viability of the cells. For example, among the epithelial cell lines, Me-180 cells exhibited the highest reduction in viability with treatment (data not shown), however the housekeeping gene remained unchanged. One limitation to our study is the inherent variability between individual infections of lymphocytes which does not allow for averaging of data across independent infections. Nonetheless, our primary observations remain consistent between multiple infections, which are shown individually.

In addition to the use of small-molecule inhibitor NSC95397, another potential experimental strategy for understanding the impact of CtBP1 on persistent infection in lymphocytes is transient knock-down of CtBP1 expression using shRNA or siRNA. Primary lymphocytes and lymphocytic cell lines are notoriously challenging to transfect using lipid-based approaches [81], but electroporation has been used successfully to deliver regulatory RNA with high efficiency [82]. In our current study, we attempted to transfect our persistently-infected lymphocytic cell lines with knock-down siRNA through electroporation and found that electroporation alone was sufficient to upregulate viral gene expression (data not shown). Future attempts to use a CtBP1 knock-down approach may include stable transduction with an inducible shRNA expression vector prior to infection of the lymphocytes, which would allow controlled expression of the regulatory RNA and resulting CtBP1 knock-down only after the persistent phase of infection has been established.

CtBP gene regulation is complex with many paradoxical activities reported for its function. The differences in CtBP expression profile between our cell line models of lytic and persistent infection suggest that distinctions in known function, structure, and localization of the two CtBP homologs may be important for infection outcome in these cells. While CtBP1 is ubiquitously expressed, CtBP2 expression is more tissue and cell-type specific [29]. Structurally, CtBP1 and CtBP2 differ slightly by a nuclear localization signal (NLS) only present in the N-terminal of CtBP2 and a PDZ-binding domain only present in the C-terminal of CtBP1 [83]. The NLS present, and a key p300 acetylation site on lysine 10 within the NLS, are responsible for the nuclear localization of CtBP2 [45]. On the other hand, the localization of CtBP1, which is found both in the cytoplasm and the nucleus, is subject to more complex regulation; sumoylation at lysine K428, in conjunction with the PDZ-binding domain regulate nuclear localization [83]. CtBP1 can also be recruited to the nucleus by a CtBP2-dependent mechanism [84]. Additionally, distribution of CtBP1 between the cytoplasm and the nucleus is also reported to be dependent upon the cell-type, further implicating other factors in localization regulation [83–86]. How these reported differences in the complex regulation of CtBP impact the viral life cycle in these cells will require additional study.

This is the first investigation into a possible role for CtBP in persistent infection of lymphocytes, and we observed that NSC95397 treatment could release a CtBP-associated repression of *E1A* in infected T cell lines. Although the Jurkat infections are lytic and KE37 infections persist for months, both show suppression of infection kinetics relative to epithelial cells [17]. A549 cells produce high levels of viral late proteins within 24 h of infection, while Jurkat and KE37 do not achieve peak levels until 1–3 or 3–7 dpi, respectively, despite equivalent amounts of viral DNA (Fig. 1a and [17, 21]). Transcription is also repressed in both cell lines relative to A549 (Fig. 1b). Whether these overall reduced levels of viral transcripts stem from a repressive mechanism at the *E1A* promoter remains to be determined, but it seems likely that repression of the master regulator of AdV infection, *E1A*, could have a profound influence on the infection dynamics. We were surprised to find that, under the same treatment conditions, we observed no de-repression of *E1A* in BJAB cells. It is possible that the binding partners incorporated into CtBP complexes between our B and T cell lines may be different, and additionally, may be influenced by the differences in basal NAD^+^/NADH ratios detected in our lymphocyte cell lines [35]. These are all areas worthy of further investigation.

In one of the only other reports of a direct mechanism involved in establishment of persistent infection, Zheng et al. showed that repression of AdV transcription, resulting from interferon (IFN) α- and IFNγ-induced recruitment of E2F/Rb complexes to the *E1A* enhancer, was able to induce persistent infection in primary and normal epithelial cells [87]. While IFN-treatment allowed epithelial cells to survive infection for long periods of time with reduced viral gene expression in this study, production of infectious virus could be detected at all time points [87]. Notably, upon cessation of IFN-treatment, viral replication rebounded dramatically [87]. In contrast, in both naturally-infected lymphocytes extracted from tonsil and adenoid tissue and in lymphocyte cell lines, viral transcription is similarly repressed but infectious virus can be detected only in rare instances [2, 17]. This suggests that, even without chronic IFN exposure, a more extensive repression of viral gene expression has occurred in lymphocytes than what was described for IFN-treated epithelial cells. Whether the IFN-E2F/Rb axis contributes to persistent infection in lymphocytes has not been determined, but different and/or additional mechanisms of transcriptional repression likely regulate persistence in lymphocytes.

Other mechanisms of viral transcriptional repression have been reported in AdV infection of epithelial cells that potentially link the metabolic state of the cell to regulation of persistent infection through NAD-dependent enzymes. Sirtuins (NAD^+^-dependent Class III HDACs) have been implicated in regulation of AdV gene expression. Silencing RNA (siRNA) knockdown of all seven human sirtuins (SIRT1–7) has been shown to increase AdV-C5 titers by 1.5- to 3-fold [88]. In the same vein, activation of sirtuins through resveratrol treatment inhibits adenovirus DNA replication [89, 90]. Another NAD^+^-dependent enzyme to have been studied in lytic infection is Poly (ADP-Ribose) Polymerase 1 (PARP1); the AdV E4orf4 protein has been found to increase production of viral progeny through inhibition of PARP1, which is activated by the infection-induced DNA damage response (DDR) [91]. PARP-induced synthesis and attachment of long poly (ADP-ribose) chains to proteins has been shown to regulate cellular transcription through chromatin remodeling and modification of transcription factors [92, 93]. Whether sirtuins or PARP1 contribute to the transcriptional repression of persistent infection needs further investigation.

## Conclusion

Given the unique interaction of AdV with lymphocytes, and the ubiquitous presence of AdV in the population, a more thorough understanding of the mechanisms that regulate its persistence and reactivation are needed. Overall, our data provide novel insight into metabolic factors that can influence adenoviral infections in activated human lymphocytes and reveal that modulation of the cellular NAD^+^/NADH ratio can de-repress adenovirus early and late gene expression in persistently-infected lymphocytes. Blockade of CtBP binding with its PxDLS-containing partners, including E1A, did not induce the same changes in AdV gene expression observed by direct manipulation of the NAD^+^/NADH ratios or lymphocyte activation. Thus, the increased *E1A* gene expression observed in T lymphocytes upon disruption of the CtBP interaction with PxDLS-binding partners likely represents one mechanism of a multi-factorial program of gene regulation occurring following metabolic shifts and lymphocyte activation.

## Data Availability

Data sharing not applicable to this article as no datasets were generated or analyzed during the current study.

## Abbreviations

ACTB: β-actin
ADP: Adenovirus Death Protein
AdV: Adenovirus
ALL: Acute Lymphoblastic Leukemia
BSA: Bovine Serum Albumin
CR4: Conserved Region 4
CtBP: C-terminal Binding Protein
DDR: DNA Damage Response
DMEM: Dulbecco’s modified Eagle medium
dpi: Days post infection
EIF1: Eukaryotic Translation Initiation Factor 1
FCS: Fetal Calf Serum
FITC: Fluorescein Isothiocyanate
GAPDH: Glyceraldehyde-3-phosphate Dehydrogenase
HDACs: Histone Deacetylases
HPRT1: Hypoxanthine Phosphoribosyltransferase 1
HPV: Human Papilloma Virus
HRP: Horseradish Peroxidase
IFN: Interferon
Iono: Ionomycin
MLP: Major Late Promoter
MOI: Multiplicity of Infection
NAD+: Nicotinamide adenine dinucleotide.
NADH: Nicotinamide adenine dinucleotide (NAD) + hydrogen (H)
NAM: Nicotinamide
NLS: Nuclear localization signal
PARP: Poly (ADP-Ribose) Polymerase
PBS: Phosphate-buffered saline
PE: Phycoerythrin
PFU: Plaque Forming Unit
PMA: Phorbol Myristate Acetate
RPMI: Roswell Park Memorial Institute
RT-qPCR: Reverse Transcription – quantitative Polymerase Chain Reaction
SDS-PAGE: Sodium dodecyl sulfate polyacrylamide gel electrophoresis
SF: Serum-Free
SIRT: Sirtuin
TBST: Tris-Buffered-Saline with 1% Tween
TET: Ten-Eleven Translocation
